# PLOD2 high expression associates with immune infiltration and facilitates cancer progression in osteosarcoma

**DOI:** 10.3389/fonc.2022.980390

**Published:** 2022-10-05

**Authors:** Zhen Wang, Gentao Fan, Hao Zhu, Lingfeng Yu, Diankun She, Yanting Wei, Jianhao Huang, Tianhang Li, Shoubin Zhan, Shenkai Zhou, Yan Zhu, Yicun Wang, Xi Chen, Jianning Zhao, Guangxin Zhou

**Affiliations:** ^1^ Department of Orthopaedics, Jinling Hospital, Nanjing Medical University, Nanjing, China; ^2^ Department of Orthopaedics, Affiliated Jianhu Hospital of Nantong University, Yancheng, China; ^3^ Department of Orthopaedics, Jinling Hospital, Nanjing University, Nanjing, China; ^4^ Department of Epidemiology and Biostatistics, School of Public Health, Nanjing Medical University, Nanjing, China; ^5^ Department of Orthopaedics, Jinling Hospital, Southern Medical University, Nanjing, China; ^6^ Department of Urology, Drum Tower Hospital, Nanjing University, Nanjing, China; ^7^ Jiangsu Engineering Research Center for microRNA Biology and Biotechnology, State Key Laboratory of Pharmaceutical Biotechnology, School of Life Sciences, Nanjing University, Nanjing, China

**Keywords:** PLOD2, osteosarcoma, migration, invasion, angiogenesis, immune infiltration

## Abstract

**Background:**

Osteosarcoma (OS) is the most common primary malignant bone tumors in children and adolescents. Procollagen-lysine, 2-oxoglutarate 5-dioxygenase 2 (PLOD2) is a key gene in mediating the formation of the stabilized collagen cross-link, playing an important role in the progression of cancer. However, the interaction between OS and PLOD2 has not been clarified so far.

**Methods:**

The target gene PLOD2 was screened through our own RNA-seq results and other two RNA-seq results from GEO database. The expression of PLOD2 in OS was detected by RT-qPCR, Western blot and immunohistochemistry. Functional experiments were performed to investigate the role of PLOD2 in OS cell invasion, migration and angiogenesis *in vitro*. An OS lung metastasis model was established to investigate the function of PLOD2 in OS metastasis and angiogenesis *in vivo*. The role of PLOD2 in immune infiltration in OS was explored by KEGG/GO analysis and immune infiltration analysis with TARGET, TCGA and TIMER.

**Results:**

PLOD2 was high-expressed in OS, which was related to poor prognosis of OS patients. PLOD2 promoted OS cell migration, invasion and angiogenesis *in vitro* and aggravated OS metastasis and angiogenesis *in vivo*. Bioinformatic analysis showed that PLOD2 played an important role in immune cell infiltration in OS, including CD8 positive T cells, macrophages M0 cells, DC cells, endothelial cells, iDC cells, ly endothelial cells, MEP cells, mv endothelial cells, native B cells, smooth muscle cells and Th1 cells. Immunohistochemical results showed that the expression of CD4 and CD8A was negatively correlated with the expression of PLOD2 in OS.

**Conclusion:**

PLOD2 was high-expressed in OS and promoted OS migration, invasion and angiogenesis *in vitro* and facilitated OS metastasis and angiogenesis *in vivo*. PLOD2 was associated with immune cell infiltration in OS, which could be a promising target to treat OS patients with metastasis and utilized to guide clinical immunotherapy in the future.

## Introduction

Osteosarcoma(OS) is the most common malignant bone tumors in children and adolescents, the incidence of which in the general population is 2–3/million/year worldwide (1). OS exhibits a predilection to occur in the metaphysis of long bones, and most commonly occurs in the distal femur (43%), proximal tibia (23%), or humerus (10%) (2). The most common treatments for OS include neoadjuvant chemotherapy, surgery and postoperative chemotherapy (3). With the advancements of therapeutic methods and technologies, the survival of patients with primary OS has improved to 65%-70%, but the survival of patients with metastasis or recurrent disease is only about 20% (4). So, it is important to explore the molecular mechanism underling OS metastasis.

Procollagen-lysine, 2-oxoglutarate 5-dioxygenase 2 (PLOD2) is a member of the PLOD family which is a key gene in mediating the formation of the stabilized collagen cross-link. It is known that collagen secretion and remodeling of extracellular matrix (ECM) are accelerated especially in cancer stromal cells, leading to invasion and metastasis of cancer cells (5). Therefore, PLOD2 may play an important role in cancer metastasis. Previous research showed that PLOD2 was highly expressed in various cancer types, including hepatocellular carcinoma (HCC), oral squamous cell carcinoma (OSCC), gastric cancer and lung cancer. In addition, the expression of PLOD2 was related to the prognosis of cancer patients (6–9; B. 10). PLOD2 was later found to be involved in immune infiltration in different cancer cell types(F. 11; Q. 12; B. 10). For instance, the expression of PLOD2 was found to be positively corelated with the activities of tumor-infiltrating immune cells(TIIC), including macrophages, neutrophils, CD4+T cells and B cells in HCC(B. 10). However, the role of PLOD2 in the progression and immune infiltration in OS remains unclear. In our research, we found that PLOD2 was highly expressed in OS. Through functional experiments we found that PLOD2 promoted OS migration, invasion and angiogenesis *in vitro* and facilitated OS metastasis and angiogenesis *in vivo*. The results of bioinformatic analysis exhibited that PLOD2 was involved in the immune infiltration in OS and was negatively correlated with the infiltration of CD4 and CD8A T cells. Our research may provide a new promising target for the clinical treatment of OS in the future.

## Materials and methods

### Sample collection

OS tissues and paired adjacent normal tissues were obtained from 22 OS patients who were admitted in Jinling Hospital (Nanjing, China) from January 2021 to January 2022. The research protocal was approved by the Ethics Committee of Jinling Hospital. All participating patients provided written informed consent authorizing the use of specimens for the intended research. All resected specimens were stored at − 80°C prior to RNA extraction.

### Cell culture

Human MG63, 143B and SJSA OS cell lines, human hFOB 1.19 osteoblast cell line and human umbilical vein endothelial cells (HUVECs) were obtained from the Cell Bank of the Chinese Academy of Sciences (Shanghai, China). All cell types were cultured in DMEM (Hyclone; Thermo Fisher Scientific, Inc.) supplemented with 10% fetal bovine serum (FBS) (Gibco; Thermo Fisher Scientific, Inc.), 100 U/ml of penicillin (Life Technologies) and 100 μg/ml streptomycin (Life Technologies) at 37°C in 5% CO_2_ and 95% air.

### Cell transfection

Three different small interfering (si)RNAs against PLOD2 were designed and synthesized by Ribobio(Guangzhou, China), and transfected into MG63 and 143B cells using Lipofectamine^®^ 2000 (Invitrogen; Thermo Fisher Scientific, Inc.). Cells were collected 48h after transfection, and the knockdown efficiency was detected by reverse transcription-quantitative PCR (RT-qPCR). The lentivirus-containing short hairpin RNA(shRNA) targeting PLOD2 was purchased from GenePharma(Shanghai, China), shRNA was transfected into 143B cell line, 48h after transfection, cells were selected with puromycin(2μg/mL) for 2 weeks to construct stable PLOD2 knockdown cell line. The sequences of PLOD2 siRNA are listed in **Table S1**.

### Immunohistochemistry

Immunohistochemistry (IHC) was performed according to the manufacturer’s constructions. The sample was deparaffinized with xylene, rehydrated with ethanol, and incubated with 3% H_2_O_2_ for 5 min to block endogenous peroxidase activity. Then, antigen retrieval was performed by incubating the samples with sodium citrate buffer (pH 6.0) for 20 min at 95°C. After blocking with 5% normal goat serum for 10 min at 20°C, the sections were incubated with polyclonal antibodies against PLOD2 (1:2000, CST), VEGF (1:500, Servicebio, Wuhan, China), CD31(1:500, Servicebio, Wuhan, China), CD4 (1:500, Servicebio, Wuhan, China) and CD8A (1:500, Servicebio, Wuhan, China) at 4°C overnight and then incubated with secondary antibodies (1:200, Servicebio, Wuhan, China). The images were captured with the Olympus FSX100 microscope (Olympus,Japan).

### Picrosirius red staining

Picrosirius red staining was used for histological visualization of collagen fibers. Picrosirius red (Servicebio, Wuhan, China)) was used to quantify the percentage of collagen fibers in OS specimens and metastatic tumors in the lung. All the samples were formalin-fixed, paraffin-embedded, cut into sections, and stained with picrosirius red. The sections were captured by Olympus FSX100 microscope (Olympus, Japan).

### RNA extraction and RT-qPCR

Total RNA was extracted from the cultured cells and tissues using Trizol Reagent (Invitrogen, CA, USA) according to the manufacturer’s instruction. For the quantification of mRNA, 1 μg of total RNA obtained from cultured cells or tissues was reverse-transcribed to cDNA using HiScript III 1st Strand cDNA Synthesis Kit (Vazyme, Nanjing, China). RT-qPCR was performed using SYBR Green dye (Vazyme) with LightCycler96 System (Roche, IN, USA). All reactions were run in triplicate. After the reactions were complete, the cycle threshold (CT) values were determined with the LightCycler96 software. A comparative CT method was used to compare each condition to the control reactions. GAPDH was used as an internal control, and the relative level was calculated with the equation 2^−ΔΔCT^. The PLOD2 and GAPDH primers are listed in **Table S2**.

### Western blot

All proteins were extracted through radio-immunoprecipitation assay (RIPA) (Beyotime, Shanghai, China) supplemented with PMSF. The extracted protein lysates were separated in 10% SDS-polyacryl-amide gel electrophoresis (SDS-PAGE) and transferred to 0.22 μm PVDF membranes (Millipore, Massachusetts, USA). The membrane was sealed with 5% skim milk and then incubated with primary antibodies at 4°C overnight. Then, the membrane was washed with 1× TBST buffer for 15 min×3. The secondary antibodies were incubated for 1 h at room temperature, and washed with 1× TBST buffer for 15 min×3. Then, the membrane was incubated with ECL substrate (Thermo Fisher, CA, USA) according to the manufacturer’s instructions and the bands were detected with the SuperSignal West Pico chemiluminescence substrate (Pierce, Thermo Scientific). The protein bands were analyzed with ImageJ (NIH).

### Wound healing assay

Wound healing assays were performed to evaluate the migration ability of OS cells. After 24-h transfection, MG63 and 143B cells were seeded into six-well plates (6 × 10^^5^ cells/well). After 48-h seeding, pipette tips (200 μl) were used to scrape a straight scratch in the confluent cell layer and then cultured in 2% fetal bovine serum (FBS) medium. After washing the cells with PBS to remove cellular fragments, each wound was imaged at 0 and 24 h under the microscope (EVOS M7000; Thermo Fisher) at 100×. Cell migration was quantified by measuring the relative wound areas with ImageJ (NIH).

### Transwell assay

For the transwell assay, cells suspended with serum-free DMEM medium were seeded into the upper transwell chamber (Corning) after 24-h transfection. 20% FBS DMEM medium was filled in the wells of the lower chamber. After 36-h culture, the filters were fixed in methanol and stained with 0.1% crystal violet. The upper cells of the filters were gently abraded, and the lower cells migrated across the filters were imaged and counted under the microscope (EVOS M7000; Thermo Fisher) at 100×. The numbers of migrated cells were counted and calculated by ImageJ (NIH).

### Tube formation assay

HUVECs were seeded into 96-well plates with 100 μL polymerized Matrigel (BD Biosciences, San Jose, CA, USA) and then co-cultured with the supernatant of OS cells at 37°C for 24 h. Cells were then stained with calcein (5μM) (Yeasen, Shanghai, China) (13). The capillary-like structures were photographed under the microscope (EVOS M7000; Thermo Fisher) at 100× and the number of branches was analyzed by ImageJ (NIH)

### Animal experiments

Six 4-week-old male BALB/c nude mice (Model Animal Research Center of Nanjing University, Nanjing, China) were equally randomized into two groups. 143B cells (1 × 10^^5^) transfected with a lentiviral vector sh-PLOD2 or sh-NC were injected into the tail veins of mice (three mice per group). Lung metastasis was monitored with a Xenogen IVIS Spectrum Imaging System (PerkinElmer, USA). After 8 weeks, the lungs of mice were excised under anesthesia, and the metastatic tumors in the lung were marked and the lung metastatic nodules were validated using hematoxylin-eosin (HE) staining by microscopy.

### Public database

The gene expression profiles from GSE16088 (including 14 OS samples and 6 normal samples; platform: GPL96) and GSE19276 (including 20 high level malignancy OS samples and 5 OS samples with favorable prognosis; platform: GPL6848) were obtained from GEO database (http://www.ncbi.nih.gov/geo). The gene expression and clinical data of OS patients were from the TARGET (https://ocg.cancer.gov/programs/target) database. And we performed the pan-cancer analysis with GEPIA (http://gepia.cancer-pku.cn/).

### Differential expression genes

A total of 98 OS patients from TARGET database were equally divided into PLOD2 relatively high expression group and low expression group based on the median expression. DEGs between the two groups was determined by Package ‘limma’. “Adjusted P < 0.05 and Log_2_(Fold Change) >1.3 or Log_2_(Fold Change) < −1.3” were defined as the threshold for the differential expression of mRNAs.

### KEGG enrichment and GO analysis

Cluster Profiler package (version: 3.18.0) in R was employed to analyze the enrichment of KEGG pathway and GO function of potential targets. The R software ggplot2 package was used to draw boxplot; the R software pheatmap package was used to draw heatmap.

### Immune infiltration analysis

TIMER (https://cistrome.shinyapps.io/timer/) is an intuitive software used to systematically evaluate the infiltration of various immune cells and their clinical impacts (14). In this study, we examined the expression of PLOD2 in different cancer types with TIMER DiffExp module, detected the expression of PLOD2 in sarcoma, and analyzed the correlation of tumor purity and tumor-infiltrating immune cells with TIMER Gene module and Correlation module. Differences in immune subtype between PLOD2 high-expressed and low-expressed groups were analyzed by Immune Subtype Classifier and xgboost R package. The “CIBERSORT” R package was used to analyze the profiles of 22 tumor-infiltrating immune cells in OS patients and the differential infiltrated immune cells between these two groups (15). The “xCell” R package was utilized to analyze more kinds of differential infiltrated immune cells between these two groups (16).

### Statistical analysis

All date were expressed as the mean ± standard deviation (SD). Statistical analyses were performed using Prism software (GraphPad Software 8), and consisted of analysis of variance followed by Student’s t-test when comparing two experimental groups. One-way ANOVA analysis was used to compare the differences between groups. The correlation between PLOD2 expression and clinicopathological variables was calculated by the chi-square test. Overall survival (OS), progression-free survival (PFS) and disease-specific survival (DSS) were determined with the Kaplan-Meier method. All experiments were triplicated, and P < 0.05 was considered statistically significant.

## Results

### PLOD2 is highly expressed in OS cells and tissues and is related with prognosis of OS patients

**Figure 1:**
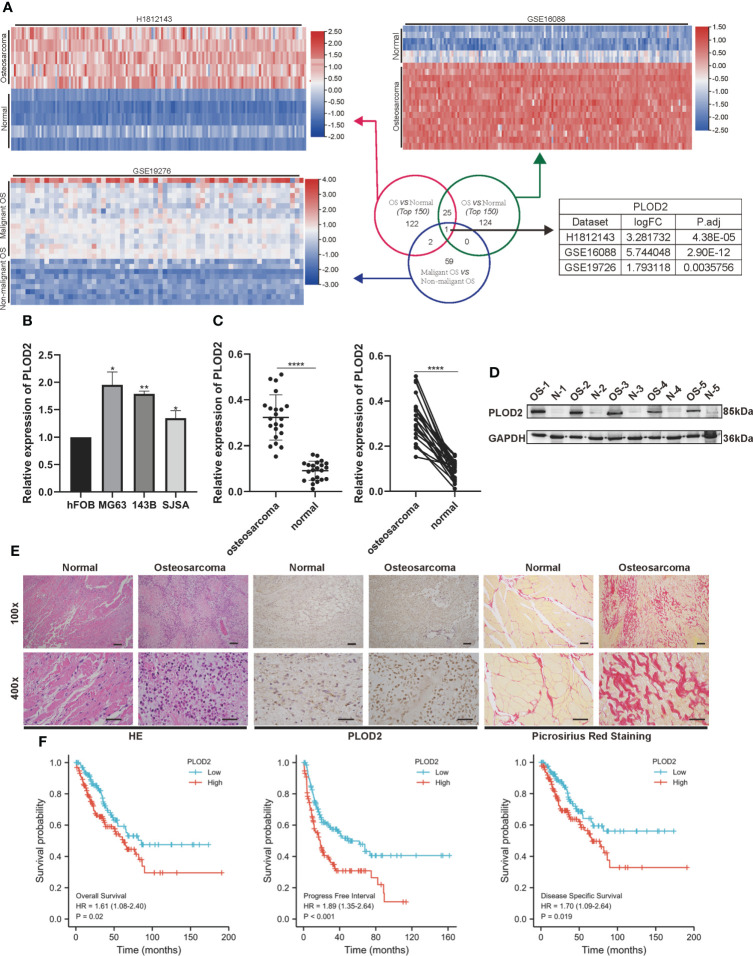
PLOD2 is highly expressed in OS cells and tissues and is related with prognosis of OS patients. **(A)** Venn diagram showing the overlap of the target genes from three RNA-seq results (GSE16088, GSE19276, H1812143) with logFC>1.5 and P.adj<0.05. **(B)** The expression of PLOD2 in OS cell lines (MG63, 143B, SJSA) compared with normal cell line hFOB. **(C)** Detection of the relative expression of PLOD2 by RT-qPCR in 22 OS tissues and adjacent normal tissues. **(D)** Detection of the relative expression of PLOD2 by Western blot in 5 OS tissues and adjacent normal tissues. **(E)** HE, IHC and Picrosirius Red Staining of OS and adjacent normal tissues. The expression of PLOD2 was analyzed based on IHC staining. The samples were imaged at 100× magnification, Scale bar = 100 μm and 400× magnification, Scale bar = 50 μm. **(F)** Kaplan-Meier analyses of the overall survival, progress free interval and disease specific survival of OS patients with high and low expression levels of PLOD2. All data are presented as the means ± SD, *P < 0.05, **P < 0.01, ****P < 0.0001. (student’s t test)'.

Firstly, we performed RNA-seq with 5 pairs of OS and adjacent normal tissues(H1812143), screened the up-regulated genes with logFC>1.5 and P.adj<0.05, and identified 150 top genes with heatmap. Then, utilizing two RNA-seq results from GEO database GSE16088 (including 14 OS samples and 6 normal samples) and GSE19276 (including 20 high level malignancy OS samples and 5 OS samples with favorable prognosis), we filtered the up-regulated genes with logFC>1.5 and P.adj<0.05 and presented 150 top genes and 62 top genes with the heatmap. Finally, we took the intersection among these three RNA-seq results: PLOD2 **(** **Figure 1A** **)**. Pan-cancer analysis showed that PLOD2 was highly expressed in different cancer types **(** **Figures S1A, B** **)**. Then, we examined the expression of PLOD2 in OS cell lines and OS tissues, and found that PLOD2 was highly expressed in MG63, 143B and SJSA cell lines compared with hFOB cell line **(** **Figure 1B** **)**. Then, we detected the expression of PLOD2 in OS and adjacent normal tissues obtained from Jinling Hospital, and found that PLOD2 was highly expressed in OS tissues compared with that in the adjacent normal tissues by RT-qPCR **(** **Figure 1C** **)** and western blot **(** **Figure 1D** **)**. In addition, the baseline information of 22 osteosarcoma patients have been listed and the high expression of PLOD2 was correlated with patients’ Enneking stage and distant metastasis **(** **Table 1** **)**. Hematoxylin-eosin (HE) staining showed the pathological features of OS and adjacent normal connective tissues, and IHC results showed that PLOD2 was highly expressed in OS tissues as compared with that in the adjacent normal tissues **(** **Figure 1E** **)**. It was reported that PLOD2 participated in synthesis of type I collagen (17). So, we conducted the Picrosirius Red Straining and the result showed that OS tissues contained more type I collagen than normal tissues **(** **Figure 1E** **)**, suggesting that PLOD2 was highly expressed in OS tissues. Then, we analyzed the prognosis of OS patients with different levels of PLOD2 expression, and found that high expression of PLOD2 was correlated with low overall survival (OS), low progression-free interval (PFI) and low disease-specific survival (DSS) **(** **Figure 1F** **)**, suggesting that PLOD2 may play an essential role in OS progression.

**Table 1:** The relationship between clinicopathological features and PLOD2 expression in 22 osteosarcoma patients.

Parameters	Total number	PLOD2 expression		
		Low (n=11) high (n=11) p Value		
Gender				
Male	13	6	7	
Female	9	5	4	0.665
Age				
<18	19	9	10	
≥18	3	2	1	0.534
Enneking stage				
I + IIA	12	9	3	
IIB/III	10	2	8	^*^ 0.01
Tumor size				
≤ 5	12	7	5	
> 5	10	4	6	0.392
Location				
Tibia/femur	14	8	6	
Elsewhere	8	3	5	0.375
Metastasis				
Yes	12	10	2	
No	10	1	9	^**^ 0.001
Total	22	11	11	** **

*p < 0.05, **p < 0.01, chi-square test.

### PLOD2 promotes OS cell migration, invasion and angiogenesis *in vitro*

**Figure 2:**
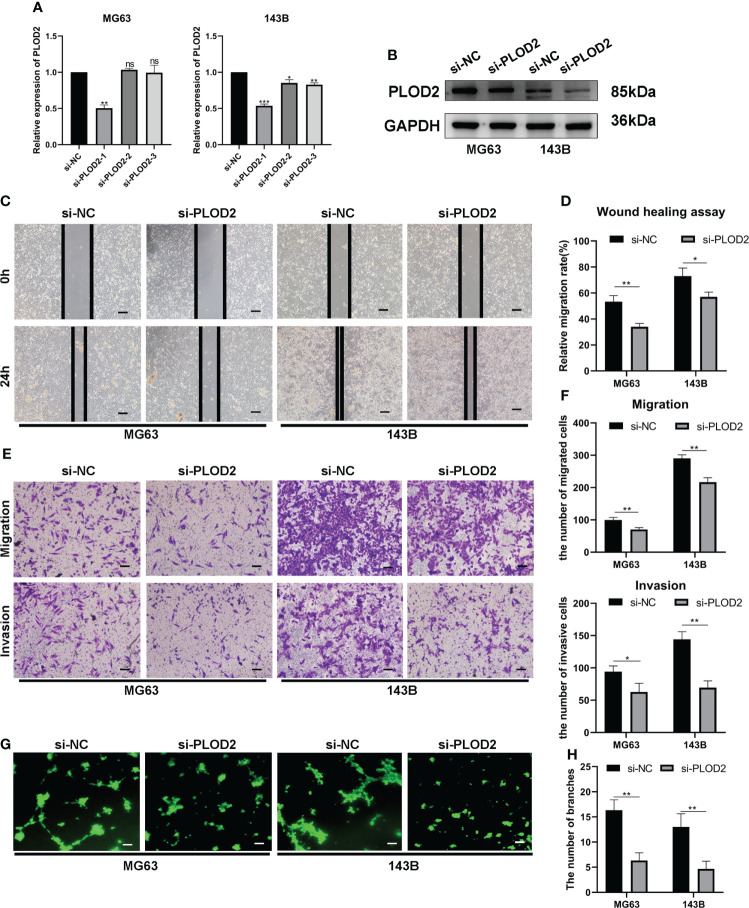
PLOD2 promotes OS migration, invasion and angiogenesis *in vitro*. **(A)** The expression of PLOD2 was measured by RT-qPCR in MG63 and 143B cell lines after transfecting three PLOD2 siRNA. **(B)** The expression of PLOD2 was detected by western blot in MG63 and 143B cell lines after transfecting si-NC and si-PLOD2. **(C, D)**. The wound healing assays were performed to assess OS cell migration ability. The samples were imaged at 100× magnification. Scale bar = 100 μm. **(E, F)**. Transwell assays were performed to assess the migration and invasion ability of OS cells. The samples were imaged at 100× magnification. Scale bar = 100 μm. **(G, H)**. Tube formation assays were used to investigate the effect of PLOD2 in MG63 and 143B cells on human umbilical vein endothelial cells (HUVECs). The samples were imaged at 100× magnification. Scale bar = 100 μm. All data are presented as the means ± SD, *P < 0.05, **P < 0.01, ***P < 0.001. (student’s t test).

Then, we planned to explore the function of PLOD2 in OS. Firstly, we transfected three PLOD2 siRNA into MG63 and 143B cell lines, and found that si-PLOD2-1 knocked down the expression of PLOD2 significantly **(** **Figures 2A, B** **)**, so we selected si-PLOD2-1 for further research. Based on the previous research results (18, 19), we inferred that PLOD2 may play an important role in OS metastasis and angiogenesis, and therefore we examined the migration and invasion ability of OS cells by wound healing assay and transwell assay. The results manifested that the migration and invasion ability of OS cells were reduced in si-PLOD2 group as compared with si-NC group **(** **Figures 2C–F** **)**. To determine the role of PLOD2 in tumor angiogenesis, we performed the tube formation assay, and the results showed that more capillary tubule formation was observed in si-NC group than si-PLOD2 group **(** **Figures 2G, H** **)**. These results exhibited that PLOD2 promoted OS cell migration, invasion and angiogenesis *in vitro*.

### PLOD2 facilitates OS metastasis and angiogenesis *in vivo*

**Figure 3:**
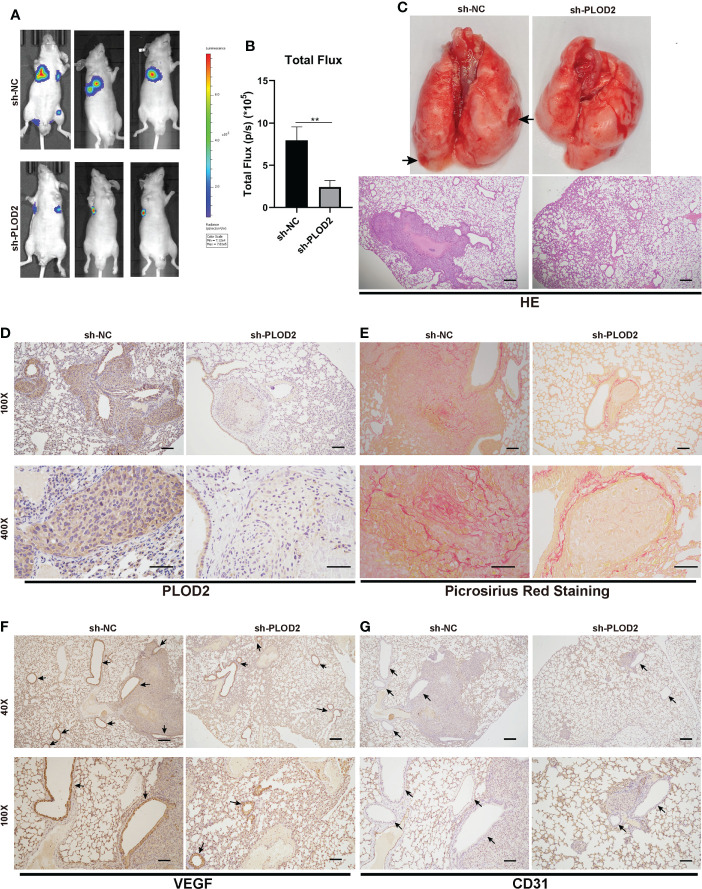
PLOD2 promotes OS metastasis and angiogenesis *in vivo*. **(A, B)**. 143B cells transfected with a lentiviral vector sh-PLOD2 or sh-NC were injected into nude mice *via* the tail vein (1 × 10^5^ cells per mice, n = 3 each group). Representative image and analysis of luminescence intensity in tail vein-injected mouse models. **(C)**. The lung specimen and HE staining of metastatic lung nodules. The HE staining samples were imaged at 40× magnification. Scale bar = 250 μm. **(D)**. IHC of metastatic lung nodules in sh-NC and sh-PLOD2 group, the expression of PLOD2 was analyzed based on IHC results. The samples were imaged at 100× magnification, Scale bar = 100 μm and 400× magnification, Scale bar = 50 μm. **(E)**. Picrosirius Red Staining of metastatic tumor in the lung. The samples were imaged at 100× magnification, Scale bar = 100 μm and 400× magnification, Scale bar = 50 μm. **(F, G)**. IHC of metastatic tumors in the lung. The small blood vessels were marked with black arrows. The expression of angiogenesis protein VEGF and CD31 was analyzed. The samples were imaged at 40× magnification, Scale bar = 250 μm and 100× magnification, Scale bar = 100 μm. All data are presented as the means ± SD, **P < 0.01. (student’s t test).

To determine the role of PLOD2 in tumor metastasis, we established an OS lung metastasis model. Firstly, 143B cells were transduced with lentivirus sh-PLOD2 and lentivirus sh-NC were injected into male nude mice *via* the tail veins. It was found that the fluorescence intensity in the lung in sh-PLOD2 group was significantly lower than that in sh-NC group **(** **Figures 3A, B** **)**. The lung gross specimen and HE staining showed that the metastatic nodules in sh-NC group were more apparent than those in sh-PLOD2 group **(** **Figure 3C** **)**. Then, the protein expression level of PLOD2 in the lung was detected by IHC. The results showed that the expression of PLOD2 in sh-PLOD2 group was significantly down-regulated **(** **Figure 3D** **)**. Picrosirius Red Staining of the lung tissue showed more type 1 collagen in sh-NC group than that in sh-PLOD2 group **(** **Figure 3E** **)**. Considering the role of PLOD2 in tumor angiogenesis *in vitro*, we performed IHC of angiogenesis related protein which including VEGF and CD31. The results showed that more small blood vessels formed in sh-NC group than those in sh-PLOD2 group **(** **Figures 3F, G** **)**. All these results indicate that PLOD2 promoted OS metastasis and angiogenesis *in vivo*.

### DEGs in PLOD2 high- and low-expressed groups and functional enrichment with KEGG and GO analysis

**Figure 4:**
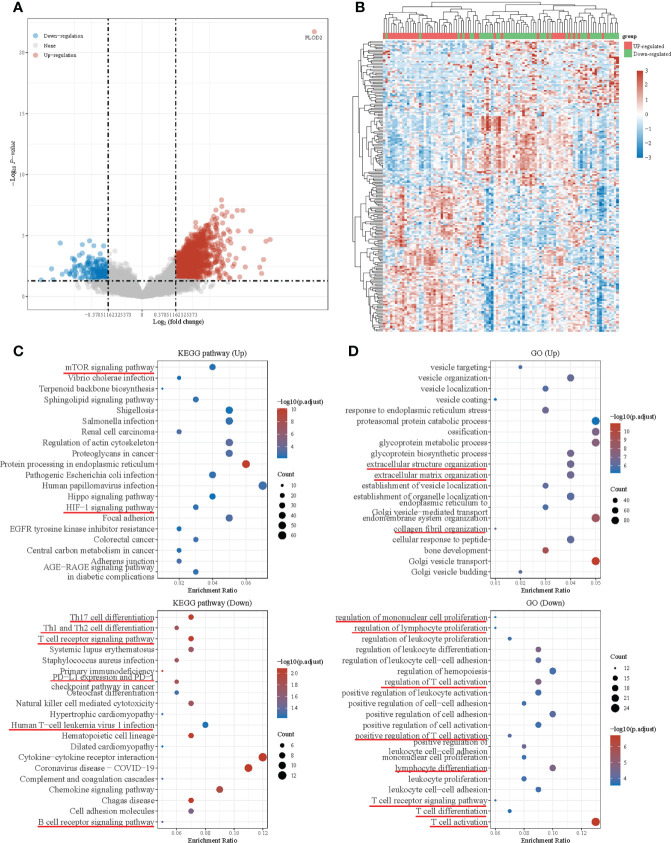
Differential expressed genes in PLOD2 high-expressed and low-expressed groups and functional enrichment with KEGG and GO analysis. **(A)** Volcano plot: The volcano plot was constructed using the fold change values and P-adjust. Red dots indicate up-regulated genes; blue dots indicate down-regulated genes; grey dots indicate not significant. **(B)** Heatmap: The heatmap of the differential gene expression, different colors represent the trend of gene expression in different tissues. The top 50 up-regulated genes and top 50 down-regulated genes were showed in this figure. **(C)** Functional enrichment: The enriched KEGG signaling pathways were selected to demonstrate the primary biological actions of major potential mRNA. The abscissa indicates gene ratio and the enriched pathways were presented in the ordinate. **(D)** Gene ontology (GO) analysis of potential targets of mRNAs. The biological process (BP), cellular component (CC), and molecular function (MF) of potential targets were clustered based on ClusterProfiler package in R software (version: 3.18.0). Colors represent the significance of differential enrichment, the size of the circles represents the number of genes, the larger the circle, the greater the number of genes. In the enrichment result, P <0.05 or FDR <0.05 is considered to be a meaningful pathway (enrichment score with −log10 (*P*) of more than 1.3).

Then, we downloaded the data of 98 OS patients from TARGET database for further analysis. Firstly, we equally divided them into a PLOD2 high-expression group (n=49) and a low expression group (n=49) based on the median expression of PLOD2. Then, we analyzed DEGs between the two groups and presented the results in a volcano plot and heatmap **(** **Figures 4A, B** **)**. KEGG pathway enrichment analysis and GO analysis were performed based on DEGs between PLOD2 up-regulated group and PLOD2 down-regulated group. It was found that mTOR signal pathway and HIF-1 signal pathway were activated in PLOD2 up-regulated group, which played important role in the process of angiogenesis, at the same time, extracellular structure organization pathway, extracellular matrix organization pathway and collagen fibril organization pathway were also activated, which was consistent with the malignant behavior of cancer metastasis **(** **Figures 4C, D** **)**. In addition, many immune related pathways and activities were observed in PLOD2 down-regulated group, including regulation of PD-1/PD-L1 expression, activation and regulation of lymphocytes, B cells, T cells and leukocytes **(** **Figures 4C, D** **)**. These results showed that PLOD2 may act as an immune suppressor in OS.

### PLOD2 high expression associates with immune infiltration in OS

**Figure 5:**
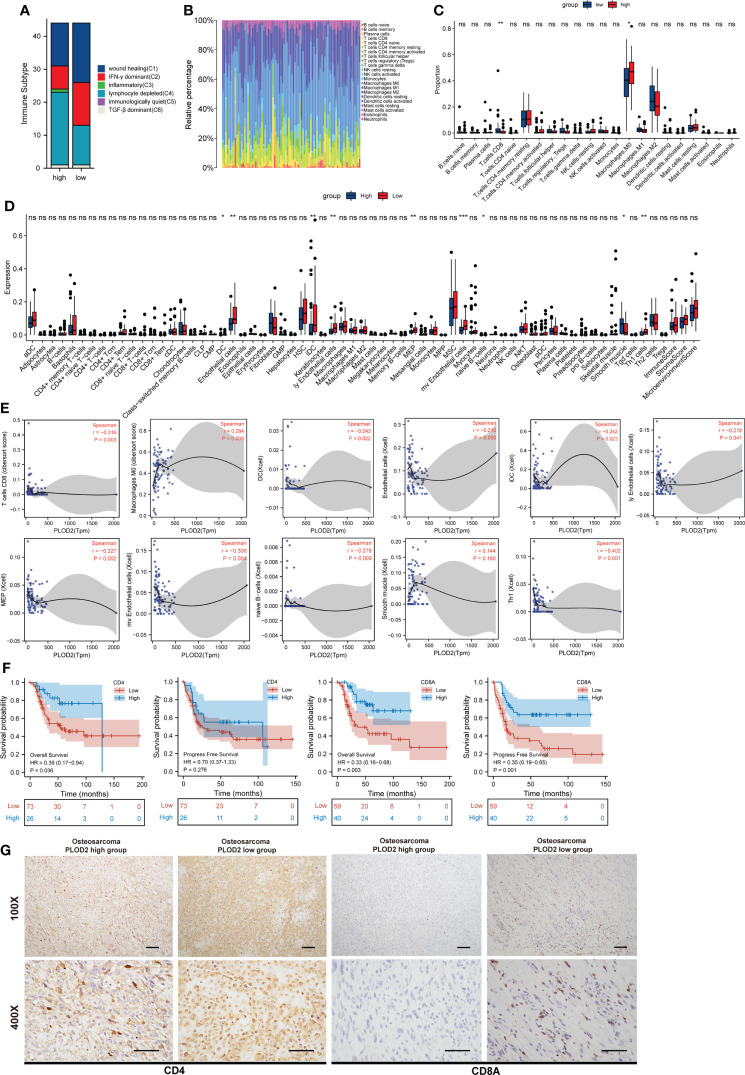
PLOD2 is associated with immune infiltration in osteosarcoma. **(A)** Immune Subtype of 88 OS patients from TARGET database between PLOD2 high-expressed group and PLOD2 low-expressed group. **(B)** The composition of immune cells among 88 OS patients from TARGET database. **(C)** Different infiltrated immune cells in PLOD2 high-expressed group and low-expressed group by CIBERSORT algorithms. **(D)** Different infiltrated immune cells in PLOD2 high-expressed group and low-expressed group by xCell algorithms. **(E)** The correlation between PLOD2 and immune cells which were differentially expressed in PLOD2 high-expressed and low-expressed group. **(F)** Kaplan-Meier analyses of the overall survival and progress free survival of OS patients with high and low expression of CD4 and CD8A. **(G)** IHC of OS tissues in PLOD2 high-expressed group and low-expressed group. The protein level of CD4 and CD8A were analyzed based on IHC results. The samples were imaged at 100× magnification, Scale bar = 100 μm and 400× magnification, Scale bar = 50 μm. All data are presented as the means ± SD, *P < 0.05, **P < 0.01, ***P < 0.001. (student’s t test).

Immune Subtype analysis showed more depleted lymphocytes and fewer IFN-γ dominant and less wound healing related immune cells in PLOD2 high-expressed group **(** **Figure 5A** **)**. Next, immune infiltration in sarcoma was analyzed by TIMER, and the results showed that the expression of PLOD2 in sarcoma was correlated with B cells, CD8 positive T cells, CD4 positive T cells, macrophages, neutrophils and DC cells **(** **Figure S1C** **)**. Survival analysis by TIMER showed that the expression of CD4 positive T cells, Neutrophil and PLOD2 were related to the clinical prognosis of sarcoma patients **(** **Figure S1D** **)**. Then, we analyzed the components of immune cells in 88 OS patients from TARGET database. Different patients exhibited different compositions of immune cells **(** **Figure 5B** **)**. To explore the immune infiltration in OS patients from TRAGET database, we equally divided them into a PLOD2 high-expressed group (n=44) and a PLOD2 low-expressed group (n=44) based on the median expression of PLOD2. As shown in **Figures 5C–E**, CD8 positive T cells and macrophages M0 cells were differentially expressed in PLOD2 high-expressed and low-expressed group with the use of CIBERSORT algorithms **(** **Figure 5C** **)**; DC cells, endothelial cells, iDC cells, ly Endothelial cells, MEP cells, mv endothelial cells, native B cells, smooth muscle cells and Th1 cells were differentially expressed in PLOD2 high-expressed and low-expressed group with the use of xCell algorithms **(** **Figure 5D** **)**. Then, we analyzed the correlation between these cells and PLOD2, finding that CD8 positive T cells, DC cells, endothelial cells, iDC cells, ly Endothelial cells, MEP cells, mv endothelial cells, native B cells and Th1 cells were negatively correlated with the expression of PLOD2, while macrophages M0 cells and smooth muscle cells were positively correlated with the expression of PLOD2 **(** **Figure 5E** **)**. All these results manifested that PLOD2 was a potent immune-related gene in OS. Combining previous consensus (20, 21), we inferred that CD4 and CD8 positive T cells played important roles in OS immune response. Thus, we laid our emphasis on CD4 and CD8 positive T cells. By analyzing the date of OS patients from TARGET database, we found that high expression of CD4 was concerned with low overall survival of OS patients and high expression of CD8A was associated with low overall survival and low progression free survival **(** **Figure 5F** **)**. Then, we performed IHC to detect the expression of CD4 and CD8A in OS tissues. The results showed that CD4 and CD8A were highly expressed in PLOD2 low-expressed OS tissues, and lowly expressed in PLOD2 high-expressed OS tissues **(** **Figure 5G** **)**. All these results suggest that PLOD2 may act as an immune suppressor in the progression of OS.

## Discussion

Osteosarcoma is one of the most malignant tumors mainly occurring in children and adolescents. Neoadjuvant chemotherapy, surgery and postoperative chemotherapy are the most common treatments currently available for OS (22). The survival of patients with primary OS is 65%-70% (4), but the survival of patients with tumor metastasis or recurrence is only 25% (23, 24). Thus, it is essential to explore the mechanism underlying OS metastasis and recurrence. Many studies have demonstrated that OS metastasis is a complex process involving multiple factors (25; C. 26). Due to the heterogeneity of OS, there are fewer accepted therapeutic strategies for OS metastasis. In this study, we performed the RNA-seq with 5 OS and adjacent normal tissues(H1812143) and screened the up-regulated top 150 genes with logFC>1.5 and P.adj<0.05. Then we utilized two RNA-seq results from GEO database: GSE16088 (including 14 OS samples and 6 normal samples) and GSE19276 (including 20 high level malignancy OS samples and 5 OS samples with favorable prognosis) with the same screening criterion. Finally, PLOD2 was selected from these RNA-seq results. PLOD2 was found as a key gene in mediating the formation of type I collagen, a major component of the tumor stroma in solid cancers (27). In addition, stabilized collagen cross-link was found playing a significant role in controlling tumor ECM stiffness(Y. 28). Previous studies have exhibited that PLOD2 was high-expressed in various types of cancer including HCC, OSCC and biliary tract cancer (9, 29; B. 10). Thus, PLOD2 may play an important role in cancer progression and metastasis. After that, many articles have reported that PLOD2 is also regulated by many other factors. For example, PLOD2 was regulated by hypoxia-inducible factor-1α (HIF-1α) to promote sarcoma metastasis (30); PLOD2 was regulated by miRNA-26a-5p and miR-26b-5p in bladder cancer (31). In this article, we demonstrated that PLOD2 was highly expressed in OS by RT-qPCR, Western blot, and IHC, and that the expression of PLOD2 was associated with the prognosis of OS patients. By functional experiments we found that PLOD2 promoted osteosarcoma migration, invasion and angiogenesis *in vitro*. With the utilization of the OS lung metastasis model, we found that PLOD2 facilitated OS metastasis and angiogenesis *in vivo*. All these results demonstrated that PLOD2 was highly expressed in different types of cancer and participated in cancer progression and metastasis.

Subsequently, we downloaded the data of 98 OS patients from TARGET database and equally divided them into a PLOD2 high-expression group (n=49) and a low expression group (n=49). KEGG and GO analysis showed that many immune related pathways were activated in PLOD2 low-expressed group, including the regulation of PD-1/PD-L1 expression and the regulation of lymphocytes, B cells, T cells and leukocytes. These results demonstrated that high expression of PLOD2 may repress immune related activities during the progression of OS thereby leading to low response in the process of immunotherapies. Today, immunotherapies have received considerable attention for their efficacy in the treatment of various tumors, and large numbers of preclinical and clinical trials have been carried out in OS (32; C. 33, 34). However, there is not much progress made in the immune treatment of osteosarcoma. After that, the immune environment of OS is mainly composed of T-lymphocytes, macrophages and other subpopulations including B-lymphocytes and mast cells (35). These immune infiltration-related cells play important role in the response of immunotherapies to OS. Thus, it is important to put our emphasis on the relationship between PLOD2 and the immune microenvironment in OS. With the application of CIBERSORT algorithms and xCell algorithms, we found that CD8 positive T cells, macrophages M0 cells, DC cells, endothelial cells, iDC cells, ly endothelial cells, MEP cells, mv endothelial cells, native B cells, smooth muscle cells and Th1 cells were differentially infiltrated between PLOD2 high-expressed group and PLOD2 low-expressed group. In addition, PLOD2 was found to be closely correlated with immune cell infiltration. Previous research revealed that CD4 and CD8 positive T cells played essential roles in immunotherapies (20, 21). Thus, we put our emphasis on the CD4 and CD8 positive T cells. Firstly, we found that the expression of CD4 and CD8A was related to the prognosis of OS patients. Then we performed IHC to analyze the expression of CD4 and CD8A in OS patients with different expressions of PLOD2, and the results showed that CD4 and CD8A were lowly expressed in PLOD2 high-expressed OS tissues, while CD4 and CD8A were highly expressed in PLOD2 low-expressed OS tissues. We conferred that the high expression of PLOD2 in osteosarcoma may repress immune cell infiltration, especially CD4 and CD8A T cells. Thus, PLOD2 may assist OS cells escaping from immune cells’ killing and aggravate OS progression in the end. All in all, PLOD2 is a key gene associated with immune cell infiltration in OS. However, more further studies are required to elucidate the detailed molecular mechanisms underlying how PLOD2 regulates immune infiltration, and further in-depth exploration is also needed to verify the clinical significance of PLOD2 in guiding immunotherapy.

## Conclusion

PLOD2 facilitated OS progression *in vitro* and *in vivo* and associated with immune infiltration in OS. PLOD2 could be a promising target to treat metastatic OS patients and could be used to guide clinical immunotherapy in the future.

## Data availability statement

The data presented in the study are deposited in the Dryad repository, accession link is https://datadryad.org/stash/share/XhKSl_T5aVR5EtYGPwYYmWBaIHvKeKhizQObXqZiSo.

## Ethics statement

The studies involving human participants were reviewed and approved by the Ethics Committee of Jinling Hospital. Written informed consent to participate in this study was provided by the participants’ legal guardian/next of kin. The animal study was reviewed and approved by the Ethics Committee of Jinling Hospital. Written informed consent was obtained from the individual(s), and minor(s)’ legal guardian/next of kin, for the publication of any potentially identifiable images or data included in this article.

## Author contributions

ZW, GF, and HZ contributed equally to this work. GZ, SBZ, XC and JZ devised the concept and designed the study. ZW, DS, YTW and SKZ carried out the most experiments. HZ, TL and LY finished the bioinformatic analysis. GF, ZW, JH, YZ and YCW contributed to the manuscript writing. All authors reviewed the manuscript and signed off on its accuracy.

## Funding

This study was supported by the Jiangsu Provincial Health Commission (NO. M2020025), Jiangsu Province Graduate Research and Innovation Program (JX22013903).

## Acknowledgments

We thank the support of Jiangsu Engineering Research Center for microRNA Biology and Biotechnology, State Key Laboratory of Pharmaceutical Biotechnology, School of Life Sciences, Nanjing University all the time.

## Conflict of interest

The authors declare that the research was conducted in the absence of any commercial or financial relationships that could be construed as a potential conflict of interest.

## Publisher’s note

All claims expressed in this article are solely those of the authors and do not necessarily represent those of their affiliated organizations, or those of the publisher, the editors and the reviewers. Any product that may be evaluated in this article, or claim that may be made by its manufacturer, is not guaranteed or endorsed by the publisher.

## Supplementary material

Supplementary Figure 1
**(A)** The expression of PLOD2 in different types of cancer in TIMER. **(B)** The expression of PLOD2 in different types of cancer in GEPIA. **(C)** The correlation between each type of TIICs (B-cells, CD4+ T-cells, CD8+ T-cells, neutrophils, macrophages, and dendritic cells) and PLOD2. **(D)** The cumulative survival of patients with low- and high- level TILCs (B-cells, CD4+ T-cells, CD8+ T-cells, neutrophils, macrophages, and dendritic cells) and PLOD2. P < 0.05 is considered statistically significant.
