# Supplementary figures and images for: PLOD2 high expression associates with immune infiltration and facilitates cancer progression in osteosarcoma
Source: Front Oncol. 2022 Oct 5;12:980390. doi: 10.3389/fonc.2022.980390 (PMC9581331; doi:10.3389/fonc.2022.980390)

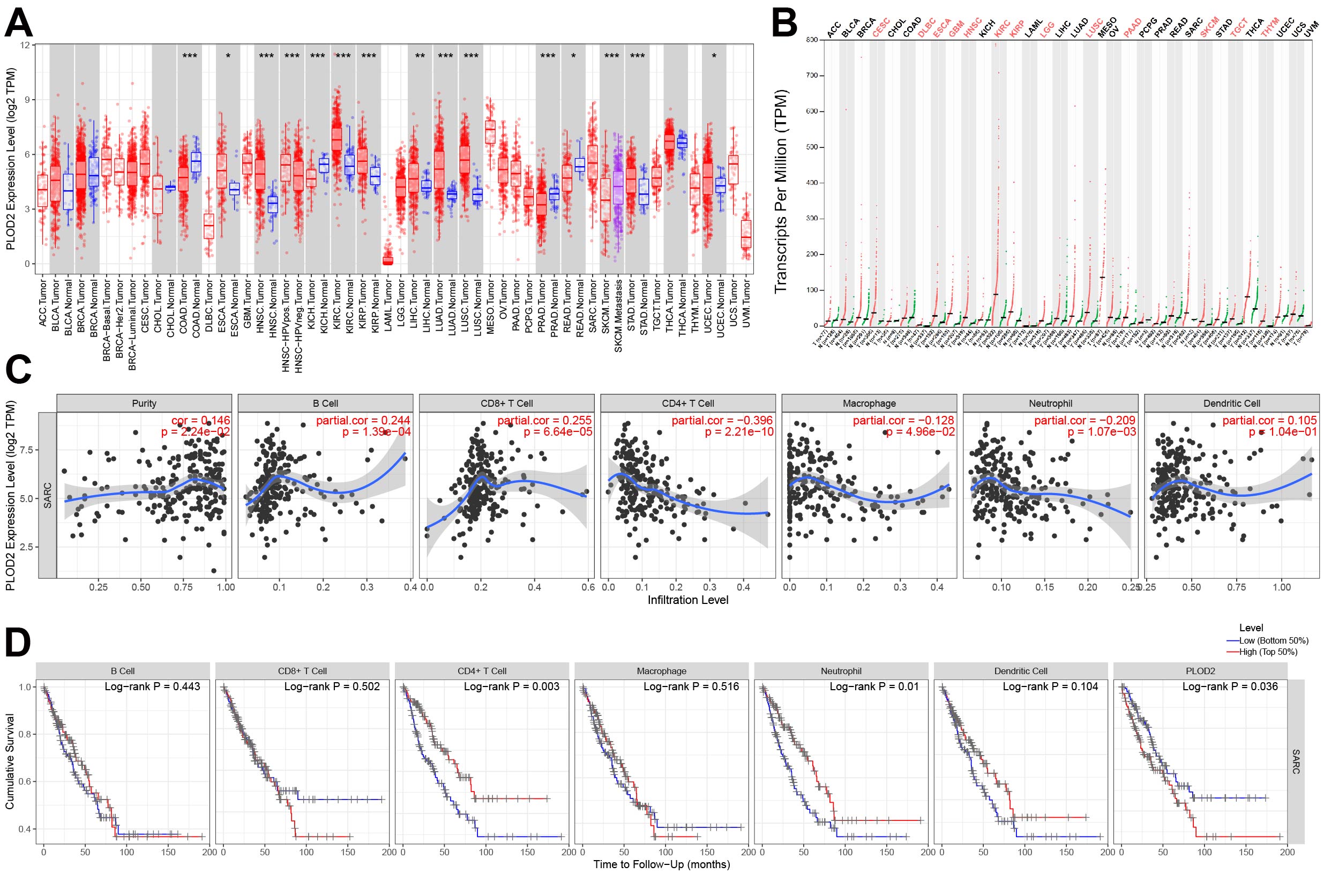

Supplement: Supplementary Figure 1 — (A) The expression of PLOD2 in different types of cancer in TIMER. (B) The expression of PLOD2 in different types of cancer in GEPIA. (C) The correlation between each type of TIICs (B-cells, CD4+ T-cells, CD8+ T-cells, neutrophils, macrophages, and dendritic cells) and PLOD2. (D) The cumulative survival of patients with low- and high- level TILCs (B-cells, CD4+ T-cells, CD8+ T-cells, neutrophils, macrophages, and dendritic cells) and PLOD2. P < 0.05 is considered statistically significant. [file Image_1.jpeg]
