# Supplementary material for: PLOD2 high expression associates with immune infiltration and facilitates cancer progression in osteosarcoma
Source: Front Oncol. 2022 Oct 5;12:980390. doi: 10.3389/fonc.2022.980390 (PMC9581331; doi:10.3389/fonc.2022.980390)
Supplement: Supplementary file 2 [file DataSheet_1.docx]

**Table S1:** **The sequences of PLOD2 siRNA**

| **si-PLOD2-1** | **GTTGCAAATTTCTAAGGTA** |
| --- | --- |
| **si-PLOD2-2** | **GAAGGTCTTTGCAGGCTAT** |
| **si-PLOD2-3** | **GGGAAACGCTATCTGAATT** |

**Table S2:** **The sequences of the primers used in the present study**

| **PLOD2 Forward primer** | **GACAGCGTTCTCTTCGTCCTCA** |
| --- | --- |
| **PLOD2 Reverse primer** | **CTCCAGCCTTTTCGTGGTGACT** |
| **GAPDH Forward primer** | **GGAGCGAGATCCCTCCAAAAT** |
| **GAPDH Reverse primer** | **GGCTGTTGTCATACTTCTCATGG** |
